# The Bluegreen Algae (AFA) Consumption over 48 h Increases the Total Number of Peripheral CD34+ Cells in Healthy Patients: Effect of Short-Term and Long-Term Nutritional Supplementation (Curcumin/AFA) on CD34+ Levels (Blood)

**DOI:** 10.3390/jpm10020049

**Published:** 2020-06-08

**Authors:** José Joaquín Merino, María Eugenia Cabaña-Muñoz, María Jesús Pelaz

**Affiliations:** 1 Dpto. Farmacologia, Farmacognosia y Botánica, Facultad de Farmacia, Universidad Complutense de Madrid, 28040 Madrid, Spain; 2 Private Dentist practice, Private Clinic, 30001 Murcia, Spain; mecjj@clinicacirom.com; 3 Stem Cell S.A Laboratory, 47151 Valladolid, Spain; mariajesus@bancostemcell.com

**Keywords:** hematopoietic stem cells (HSCs), curcumin, AFA (*Aphanizomenon flos aquae*) algae, sulforaphane, chemokines

## Abstract

Several active principles from plants could trigger the release of stem cells from the bone marrow. Stem cell mobilizers have shown side effects in patients. Thus, the purpose of this paper is to find the natural products from plants (curcuminoids, glycosinolate of sulforaphane, AFA bluegreen algae), which could be potential stem mobilizes without adverse side effects. The antioxidant curcumin [1,7-bis(4-hydroxy-3-methoxyphenyl)-1,6-heptadiene-2,5-dione], glycosinolate of sulforaphane (broccoli) or AFA (*Aphanizomenon flos*) extract promote beneficial effects in patients. The number of circulating stem cells were monitored by HSC marker-CD34 by flow cytometry in peripheral blood from healthy subjects. CD34 is a hematological stem cells (HSC) marker. A double-blind study was conducted in 22 healthy subjects. We have evaluated whether short-term AFA—*Aphanizomenon flos aquae*—algae or curcuminoids consumption (powder or liquid formulation) over 48 consecutive hours could increase the total number of peripheral CD34+ blood cells (*n* = 22, *n* = 5 subjects/group). The total number of circulating CD34+ cells were quantified after short-term and long-term nutritional supplementation; their levels were compared with their own basal levels (*n* = 5/group, controls: before taking any supplement) or placebo-treated patients (*n* = 7); their average age was 54 years old. We also evaluated whether long-term nutritional supplementation with several nutraceuticals could enhance HSC mobilization by increasing the total number of peripheral CD-34+ cell after seven or 38 consecutive days of administration (*n* = 5, with seven placebo-treated patients). The long-term administration take place with these doses/day [curcuminoids: 2000 mg/day, equivalent to 120 mg of curcuminoids/day), glycosinolate of sulforaphane (66 mg/day), plus AFA Algae bluegreen extract (400 mg/day)]. On the last day (10 a.m.) of treatment, blood samples were collected six hours after taking these supplements; the average age was 54 years old. Notably, the blue green AFA algae extract consumption over 48 h enhances HSC mobilization by increasing the total number of peripheral CD34+ cells. The long-term administration with curcuminoids, glycosinolate of sulforaphane, and AFA bluegreen algae extract also increased the total number of CD34-HSC cells after seven or 38 days of consecutive of administration in healthy subjects.

## 1. Introduction

Hematopoietic Stem cells (HSCs) are able to self-renew and differentiate into various lineages of hematopoietic progenitor cells [1,2]. It is known that certain active principles from plants could trigger the release of stem cells from the bone marrow [3]. CD34 is a marker of hematopoietic stem cells. CD34 is a transmembrane protein that recognizes the class III human progenitor cell antigen (HPCA). CD34 is exclusively expressed on certain types of immature hematopoietic colony-forming cells in bone marrow and blood, including pluripotent progenitor cells; CD45 and CD34 antibodies are each composed of mouse Ig G1 heavy chains and kappa light chains. Mobilized peripheral-blood (PB)-CD34+ have become the main source of repopulation hematopoietic stem cells (HSCs), replacing bone marrow (BM) cells for autologous/allogenic transplantation in patients with hematolymphoid diseases. The HSCs are important for bone marrow transplantation (BM) [4]. Bone marrow (BM) transplantation has achieved great success in clinical practice and has saved numerous lives but its therapeutic efficacy can be improved [5]. The classical Colony Stimulating Factor (CSF), Interleukin-8 (IL-8), and plerixafor (a CXCR4 antagonist = AMD3100) are stem cells mobilizers. Several stem cell mobilizers have been described, although many of them had associated adverse side effects in patients, such as diarrhea, nausea, pain, and thrombosis [6]. In this way, the number of circulating stem cells is a critical parameter for cell repair [4,7]. Thus, the discovery of active principles from plants as stem cells mobilizers could increase the total number of CD3+-HSC peripheral cells by triggering the release of stem cells from the bone marrow [8]. Curcumin [1,7-bis(4-hydroxy-3-methoxyphenyl)-1,6-heptadiene-2,5-dione], is the active principle from *Curcuma longa L* rhizome (family *Zingiberaceae*) [9]. Curcumin plays antioxidant, anti-inflammatory, and neuroprotective effects in patients [10,11,12]. The curcumin consumption reduces cognitive disfunction in older patients [11] and protect neurons against Alzheimer disease pathology [12]. Sulforaphane from broccoli (antioxidant) also protect neurons against brain injury [13]; furthermore, vitamin A and E can improve BM transplantation [5], whereas NAC (N-Acetil cysteine) antioxidant improved BM transplantation efficiency in immunodeficient mice (NOD/SCID) [14,15,16]. AFA bluegreen algae also had beneficial effects in patients [12].

How to select the right antioxidant for a particular patient need to be defined in future clinical trials in patients taken different antioxidants. The efficacy of a specific antioxidant for BM transplantation will largely depend of the disease type/stages and donor cell types/doses.

### Aim

The aim of this study was to investigate whether short-term and long-term nutritional supplementation (curcuminoids, glycosinolate of sulforaphane, AFA bluegreen algae) could enhance HSC mobilization in healthy subjects. We evaluated whether AFA bluegreen algae extract or curcumin (powder or liquid form) administration over 48 consecutive hours could increase the total number of peripheral CD34+ cells; their CD34+ cells were compared with their own control basal levels (before taking any supplement, *n* = 5) or placebo-treated subjects (*n* = 7).

We also evaluated whether long-term nutritional supplementation with curcuminoid [2000 mg/day, equivalent to 120 mg of curcuminoids/day), glycosinolate of sulforaphane (66 mg/day), and AFA (*Aphanizomenon flos*-bluegreen algae extract: 400 mg/day)] could enhance HSC mobilization after seven or 38 consecutive days of administration.

## 2. Material and Methods

### 2.1. Clinical Protocol for Patients

The clinical study followed a randomized study with 22 healthy subjects. Fifteen of them were short-term-treated over 48 consecutive hours (*n* = 5, AFA or *n* = 5, curcuminoids), with seven placebo-treated patients (*n* = 7). These healthy subjects consisted of 17 females and five males. The design included these study groups: five AFA (*Aphanizomenon flos*-bluegreen algae extract)-treated subjects, five subjects that consumed curcumin (*n* = 5, powder form), and five subjects that consumed liquid curcumin (*n* = 5), over 48 consecutive hours (both cases). The long-term treated patients consisted of five women (*n* = 5), and we also included seven placebo-treated subjects (*n* = 7). All patients were enrolled after providing written informed consent following the Declaration of Helsinski and updates. The average age of the patients was 54 years old and their body mass index (BMI) was normal. Their metabolic state was healthy without signs of chronic illness, allergies, blood diseases, altered digestive function, or psychiatric diseases. They were non-smokers and their sociocultural state was medium-high; 80% of them completed high school or obtained a Bachelor degree. Enrolled patients did not take any antioxidant/supplement before starting this study; they visited the clinic on the first as well as the last day of nutritional supplementation. On the last day of treatment, blood samples were collected six hours after taking curcuminoids (early morning) since curcumin reached a blood peak at this time in curcumin-treated patients [11]; 40 mL of blood were taken by an expert nurse in the extraction center. The correct nutritional supplementation was periodically checked through phone calls.

AFA blue-green extract or curcuminoids were administered over 48 consecutive hours (short-term nutritional supplementation). The long-term nutritional supplementation took place over seven consecutive days (cur 7 days) or 38 consecutive days of administration (cur 38 days). The total number of peripheral CD34+ cells were quantified after short-term (*n* = 22) or long-term nutritional administration (*n* = 5); the number of total CD34+ cells was compared with their respective controls (*n* = 5, before taking any supplement) as well as placebo-treated subjects (*n* = 7).

#### 2.1.1. Inclusion/Exclusion Criteria

This study follows the Declaration of Helsinki (1974, and updated 2000) and all enrolled healthy subjects were properly instructed before taking these supplements; they signed the appropriate consent paperwork, and all efforts have been made to minimize the number of patients. Moreover, their privacy and anonymity were also preserved at all times. The serology for HIV-1, hepatitis C and B, herpes virus, or CMV detection were negative for all patients. The total number of peripheral CD34 cells was quantified by flow cytometry after short-term or long-term nutritional supplementation. We selected patients without previous pathologies. These short-term groups are divided as detailed in the following sections.

*Short-term nutritional treated subjects (total number of enrolled patients: 22)*

- AFA bluegreen algae-treated healthy subjects over 48 consecutive hours (*n* = 5).
- Powder curcumin-treated subjects over 48 consecutive hours (*n* = 5).
- Liquid curcumin-treated subjects over 48 consecutive hours (*n* = 5)
- Placebo-treated group (*n* = 7).

AFA bluegreen extract (Klamath lage) is from Cienporciennatural company (Madrid, Spain).

Powder curcuminoids (commercial name: ERGYCARE from Nutergia company, Basque Country, Spain). 

AFA (*Aphanizomenon flos-aq*uae) bluegreen algae extract from Klamath lage (400 mg/day, *n* = 5, Cienporciennatural company, Madrid, Spain) 

Liquid curcumin (AB Micellar turmeric from AIRBIOTIC company, Madrid, Spain).

*Long-term nutritional treatments (total number of enrolled patients: fiften, plus seven placebo-treated subjects).*

**Figure 1:**
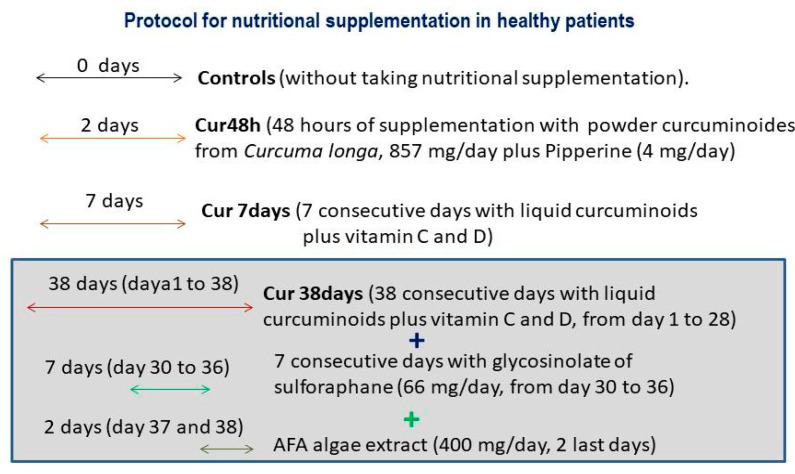
Study Groups.

- The liquid curcumin was administered over seven consecutive days (cur 7 days) or 38 consecutive days of administration in healthy subjects (cur 38 days, *n* = 5). The administration of curcumin take place in the same patient until reaching 38 days of supplementation. Thus, blood samples were collected at both selected times (seven days as well as 38 days, *n* = 5).
- Placebo-treated group (*n* = 7).
- Glycosinolate of sulforaphane (NutriSGS without C vitamin) from Cienporciennatural company (Madrid, Spain, see protocol Figure 1).

#### 2.1.2. Exclusion Criteria

Physically handicapped patients with hematological, metabolic, infective diseases, neurological/neuropsychiatry disorders (4th Edition, DSM IV) were excluded in the present study. We did not consider patients taking regular medication, stimulants, antioxidants, anticonvulsants, or atypical antipsychotic; furthermore, we excluded patients who had history of liver/kidney disease. Finally, patients prescribed with chelators or those taking antioxidants were not considered in this study. Additionally, we excluded patients who have periodontal disease or inflammatory diseases.

### 2.2. Protocol for Nutritional Supplementation in Healthy Subjects

#### 2.2.1. Short-Term Nutritional Supplementation

The *Curcuma longa* extract (liquid form) was administered over 48 consecutive hours (*n* = 5). The doses/day were as follows: [2000 mg of *Curcuma longa*, equivalent to 120 mg of curcumin/day), encapsulated with vitamin C (40 mg/day) and D (56 μg/day)].

Another group of healthy subjects consumed bluegreen AFA—Aphanizomenon flos—bluegreen algae extract over 48 consecutive hours (400 mg/day, *n* = 5). 

Two formulations of curcuminoids (*n* = 5, powder or liquid, *n* = 5) were administered over 48 consecutive hours to two different groups of healthy subjects. The powder curcumin form (ERGYCARE from Nutergia company, Basque Country, Spain) or liquid encapsulated curcumin plus vitamin C and D (from Airbiotic company, Madrid, Spain; AB micellar Turmeric) were administered as follows: 847 mg/day of *Curcuma longa* powder extract, which is equivalent to 6% of curcuminoids, 150 mg/day of NAC (N-Acetil-Cystein), plus 60 mg/day of *Pipper nigra* (pipperine). This pipperine enhances the bioavailability of curcumin at 2000% (ERGYCARE from Nutergia company, Basque Country, Spain). The total number of peripheral CD34+ cells was quantified after AFA algae or curcuminoids consumption over 48 consecutive hours; their peripheral CD34 levels were compared with their control basal levels (before taking any supplement, controls, *n* = 5) or placebo-treated subjects (*n* = 7). 

#### 2.2.2. Long-Term Nutritional Supplementation

The liquid-curcumin form was administered over 7 days (cur 7 days) or over 38 consecutive days (cur 38 days) together with several active principles from plants [2000 mg of *Curcuma longa* extract, equivalent to 120 mg of curcuminoids/day), glycosinolate of sulforaphane (NutriSGS, Madrid, 66 mg/day, administered over one week from day 31–36, included), and bluegreen AFA algae extract (the last two days of supplementation, day 37–38, 400 mg/day]. In the last day of treatment, these nutraceuticals were taken six hours before blood extraction (in the morning).

### 2.3. Protocol for Peripheral Blood Extraction

Forty milliliters of peripheral blood were extracted for total peripheral CD34 quantification in healthy patients following a modified procedure for umbilical cord processing. Briefly, a total amount of 40 mL of peripheral blood were collected in an extraction center; blood samples were transported under controlled conditions of temperature to the laboratory for HSC evaluation (Stem Cell S.A Laboratory, Valladolid, Spain). Blood samples were labelled with a blinded code to the researcher. In addition, negative serology for HIV-1, Hepatitis B and C, and herpes virus was confirmed in all blood samples. The total number of peripheral CD34+ positive cells were quantified by flow cytometry in blood. An Alicuot of 500 μL from each blood sample was taken for hemogram and quantification of peripheral CD34+ cells counts; the blood samples were isolated using a buffy coat system by adaptations of Sepax UCB HES V300 system in a flow laminar chamber (Biosafe kit). Briefly, HES was slowly (2 mL/seg) added to 20% (V/V) volume of blood, and after shaking, samples were incubated for 15 min. Samples were transferred to a Falcon tube and centrifuged at 100 r.p.m. (revolutions by minute) over six minutes (20 °C). The supernatant was transferred to a new tube and samples were taken for blood count; total number of CD34+ peripheral cells. The samples were centrifuged at 400 g for 10 min, and the supernatant was removed until reaching a final volume of 20 mL in all samples (pellet). When necessary, samples were stored at −80 °C in criotubes for further biochemical evaluations. The total nuclear cells (TNC) and white blood cells (WBC) were also measured in all samples.

### 2.4. Quantification of Total Peripheral CD34+ Positive Cells by Flow Cytometry: CD34+ Is a Marker of Hematopoietic Stem Cells (HSC) Mobilization in Patients

Whole blood samples were used for immnunophenotyping and the total number of peripheral CD34+ cells were quantified by flow cytometry in blood (BD FACSCalibur). The peripheral CD34+ counts were measured following a modified ISHAGE protocol for umbilical cord stem cells detection by flow cytometry (Stem Cell S.A Laboratory, Valladolid, Spain).

Lymphocytoid CD34+ stem cells express low levels of expression of the protein tyrosine phosphatase receptor type C enzyme CD45 (CD45dim). The changes in CD45dim CD34+ stem cell numbers could evaluate the effect of consumption of these nutraceuticals on peripheral CD34 levels in the circulation. The BD Stem reagent (CD34+/CD45+) provided in phosphate-buffered saline (PBS) contains bovine serum albumin (BSA) and 0.1% sodium azide. The reagent contains CD45 FITC, clone 2D1, and CD34 PE, clone 8G12. The kit also contains 7-AAD reagent, which is a nucleic-acid dye used to identify dead cells and 10× ammonium chloride lysing solution. This ammonium chloride solution is a fixative-free solution for red blood cell lysis. The kit contains 50 BD trucount tubes and each single tube contains a freeze-dried pellet of fluorescent beads.

The CD45 recognizes a 180 to 220 Kilodalton (kDa) human leucocyte antigen, which is a member of the leucocyte common antigen (LCA) family. The CD45 antigen is present on all human leucocytes and is weakly expressed on hematopoietic progenitor cells. BD Trucount tubes are used with this reagent. By adding the reagent and the sample directly to the BD Trucount tube, the absolute count of the cell population of interest can be directly determined (BD FACSCalibur^TM^ flow cytometer). The follows concentrations were used for antibodies.

BD Trucount tubes are used with this reagent by adding the reagent and the sample directly to the BD Trucount tube; the absolute count of the cell population of interest can be directly determined (BD FACSCalibur^TM^ flow cytometer). The selected volume for samples was 20 μL CD34/CD45, 200 μL as well as 100 μL of blood and 2000 mL of lisis buffer were used for Flow cytometry analysis (see Table 1).

### 2.5. Statistical Analysis

The Levene test confirmed a normal distribution of data. A test for equal variance as well as a normality test validated the normal distribution of data. Thus, an analysis of variance test (ANOVA) was performed in order to evaluate the effect of short-term or long-term nutritional supplementation on circulating CD34+ cells; a post-hoc Bonferroni test was used for multiple comparisons, since there was homogeneity of variance.

## 3. Results: CD34+ Hematopoyetic Stem Cell (HSC) Mobilization in Healthy Subjects

### 3.1. Protocol for Short-Term Nutritional Supplementation in Healthy Patients (Figure 2)

#### 3.1.1. AFA Bluegreen Algae Extract Consumption Increased the Total Number of Peripheral CD34+-HSC Cells without Affecting Their Circulating Levels in Curcumin–Treated Subjects

**Figure 2:**
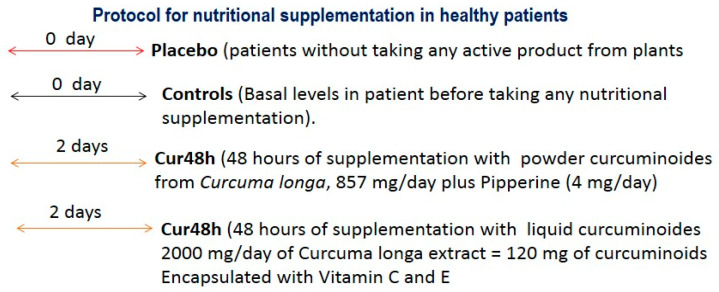
(protocol). Short-Term AFA Bluegreen Extract or Curcumin Administration over 48 Consecutive Hours with Two Different Formulations (Powder or Liquid Form).

**Figure 3:**
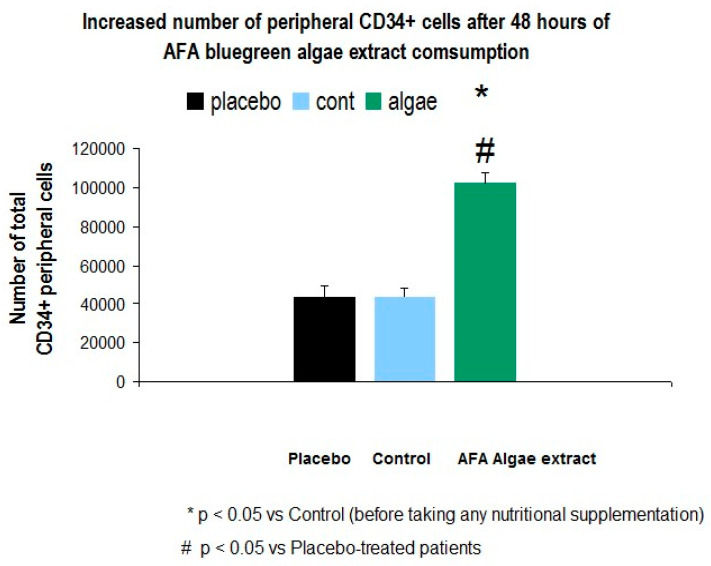
Short-term AFA supplementation over 48 h increases the total number of CD34+ peripheral cells in healthy subjects.

The ANOVA revealed a higher number of total peripheral CD34 ± HSC cells after 48 h of AFA bluegreen algae consumption in healthy subjects [F (2, 14) = 3.38, *p* < 0.001)]. The short nutritional supplementation significantly increased the total number of peripheral CD34+ cells as compared with their basal levels (*n* = 5, before taking any supplement, *p* < 0.05) or placebo-treated subjects (*n* = 7, see Figure 2 protocol- and Figure 3).

This AFA bluegreen extract is much more effective as CD34 ± HSC stem cells mobilizer than curcuminoids.

AFA blue green exact (*n* = 5): healthy subjects consumed algae AFA extract over 48 consecutive hours.

AFA blue green algae extract consumed over 48 consecutive hours (Cienporciennartural, Madrid, Spain, 400 mg/day).

Placebo (*n* = 7): patients did not receive active principles from plants.

Control (*n* = 5): total number of total peripheral CD34+ cells (basal levels: controls before taking any supplement).

#### 3.1.2. Curcumin Treatment (Powder or Liquid Forms) Did Not Increase the Total Number of CD34+ Peripheral Cells in Healthy Subjects

**Figure 4:**
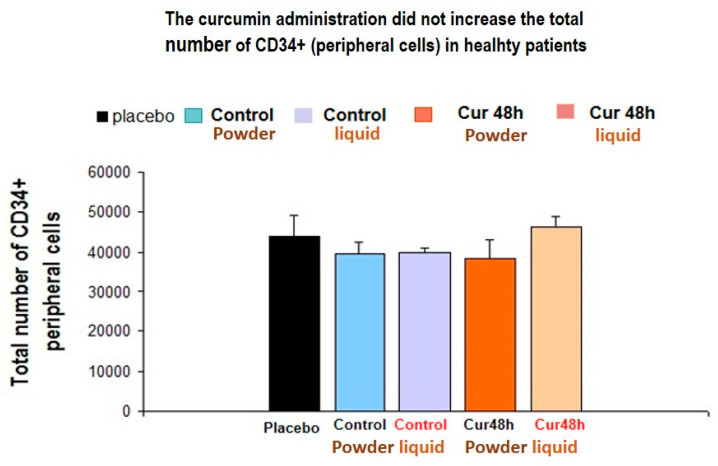
Curcumin treatment did not affect the total number of CD34+ peripheral cells in healthy subjects.

The ANOVA did not reveal a significant effect for short-term curcumin treatment in healthy subjects [F (4, 22)= 2.81, *p* = 0.65, n.s., non significant effect)]. The total circulating CD34+ cells did not differ between powder or liquid curcumin-treated groups after 48 h of consumption as compared with their controls as well as placebo-treated subjects (*p* > 0.05, n.s.). There was a trend to increase the total circulating CD34+ cells after 48 h of liquid-curcumin treatment as compared with their own control levels (*n* = 5, *p* = 0.095, n.s., before taking the supplement, see Figure 4).

Curcuminoids (powder form: ERGYCARE from Nutergia company, Spain): 857 mg/day (powder form) plus 150 mg/day of NAC (N-Acetil-cystein), plus 60 mg/day *Pipper nigra* extract (pipperine).

*Experimental groups:*

Placebo (*n* = 7): treated patients with the excipient (without active principle form plants).

Control-1 (*n* = 5): basal total number of peripheral CD34+ cells (before taking any nutritional supplementation). This is the control for curcumin in-powder form.

Cur 48 h (*n* = 5): short-term curcuminoids (powder form) consumption over 48 consecutive hours.

Control-2 (*n* = 5): basal total number of peripheral CD34+ cells (before taking any supplement). This is the control for curcumin in liquid form.

Cur 48 h (*n* = 5): curcumin was administered (in liquid form) over 48 consecutive hours.

### 3.2. The Long-Term Nutritional Supplementation with Curcumin, Glycosinolate of Sulforaphane, and AFA Bluegreen Algae Extract Increased the Total Number of CD34+ Peripheral Cells in Healthy Subjects

**Figure 5:**
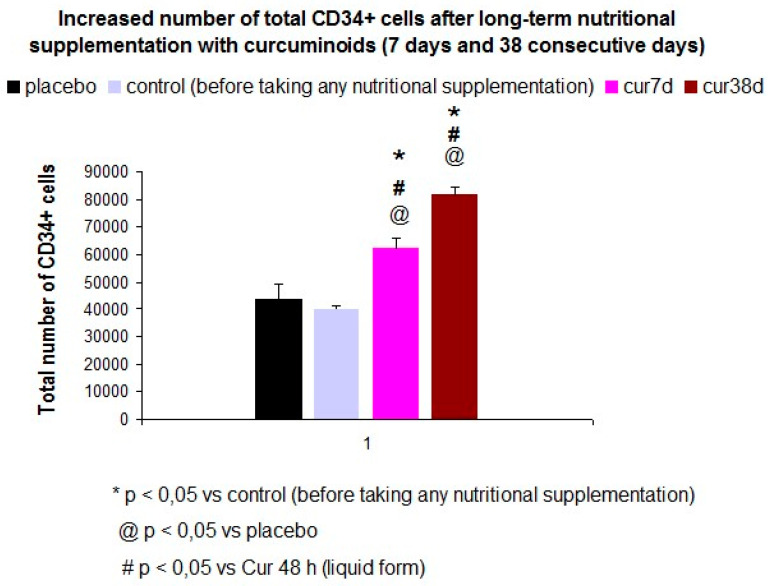
Enhanced HSC mobilization by long-term nutritional supplementation with curcumine curcumin, glycosinolate of sulforaphane in healthy subjects.

The ANOVA revealed an increased number of CD34+ peripheral cells after long-term administration in healthy subjects [F (3, 22) = 20.26, *p* < 0.001)]. This liquid-curcumin formulation enhanced HSC mobilization after seven consecutive days of administration (*p* < 0.05) but also among treated-subjects after 38 consecutive days of supplementation (*p* < 0.05). Both groups had higher peripheral CD34+ cells than placebo-treated subjects or controls (before taking any supplement, *n* = 5, *p* < 0.05 in both cases, see Figure 5).

Placebo (*n* = 7): treated-subject with the excipient. They did not consume active principles from plants.

Control (*n* = 5): total number of basal CD34+ peripheral cells (before taking any nutritional supplementation).

**Cur 7 days** (*n* = 5): curcumin (in liquid form) was administered over seven consecutive days.

*Doses (mg/day):* 2000 mg/day of *Curcuma longa* extract (a liquid form), which is equivalent to 120 mg of curcuminoids/day, plus vitamin C (40 mg/day) and D (56 μg/day). The curcumin was administered over seven consecutive days (cur 7 days).

**Cur 38 day:** 2000 mg of *Curcuma longa* extract) + glycosinolate of sulforaphane (NutrisSGS: one week of supplementation, from day 31–36, 66 mg/day) + AFA algae (in the last two days of treatment, days 37–38, 400 mg/day). These nutraceuticals were administered over 38 consecutive days.

### 3.3. Increased Number of CD34+ That Are Also CD45+ Cells, after 48 h of AFA Consumption or Curcumin (Powder Form) Administration over 24 h in Healthy Subjects

The ANOVA test revealed changes in the percentage of peripheral CD34+ cells that are also positive for CD45+ marker by short-term or long-term administration of nutraceuticals, considering all groups together [ANOVA: F (8, 46) = 5.71, *p* < 0.05, *n* = 5 subjects/group except placebo, *n* = 7]. In fact, these percentages (CD34+ and CD45+) is higher after 48 h of AFA or curcumin (powder form) consumption as compared with their respective controls (*n* = 5, before taking any supplementation, *p* < 0.05) as well as placebo-treated subjects (*n* = 7). Curcumin administration (in liquid form) over seven consecutive days (Cur 7 days) or 38 days (cur 38 days) nearly reached an effect as compared with their controls (liquid, *p* = 0.059, n.s.) or placebo-treated subjects (*p* = 0.059, n.s, see Table 2). 

### 3.4. The Number of Nucleated Cells (TNC) or White Blood Cells (WBC) Did Not Differ between Healthy-Treated Subjects

The ANOVA for TNC [F (8, 46) = 0.46, *p* = 0.87, n.s.] or WBC [ F (8, 46) = 0.63, *p* = 0.75, n.s.] did not differ at any time of treatment in healthy subjects (*p* > 0.05, all cases). The table showed mean values for each marker ± SEM (*p* > 0.05 in all cases, *n* = 5/group, see Table 3). 

Results indicated means for TNC or WBC ± SEM (*n* = 5 subjects/group) and placebo-treated subjects (*n* = 7).

**Figure 6:**
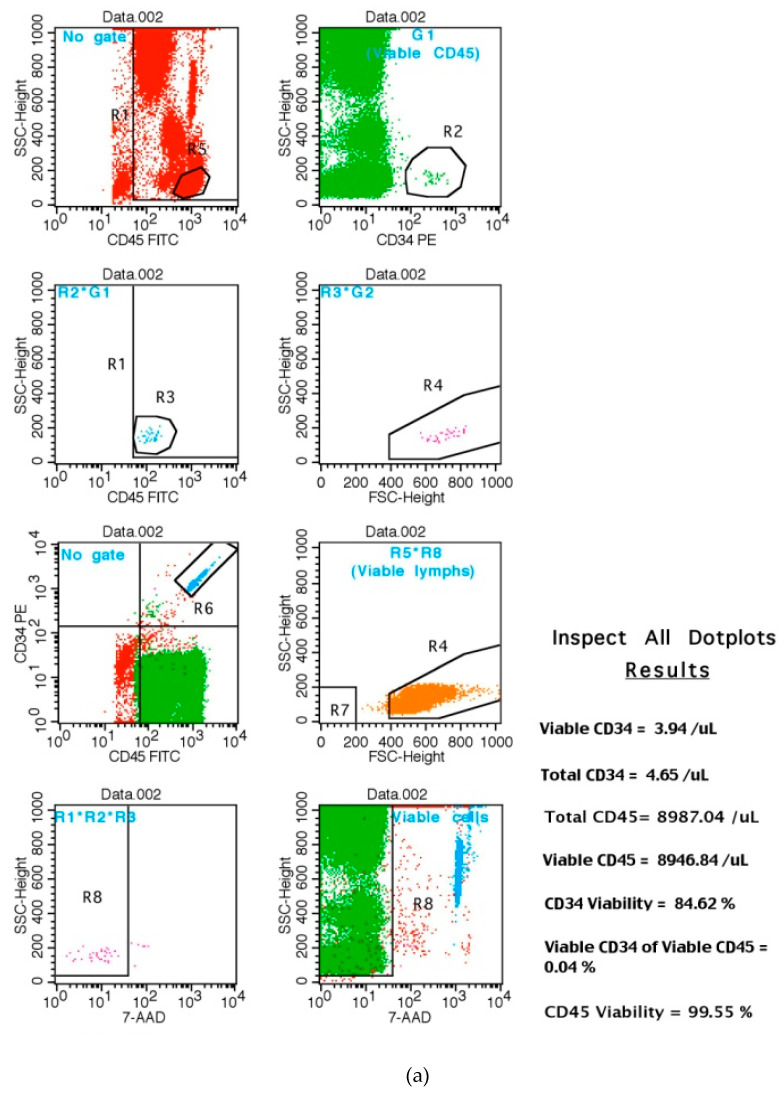

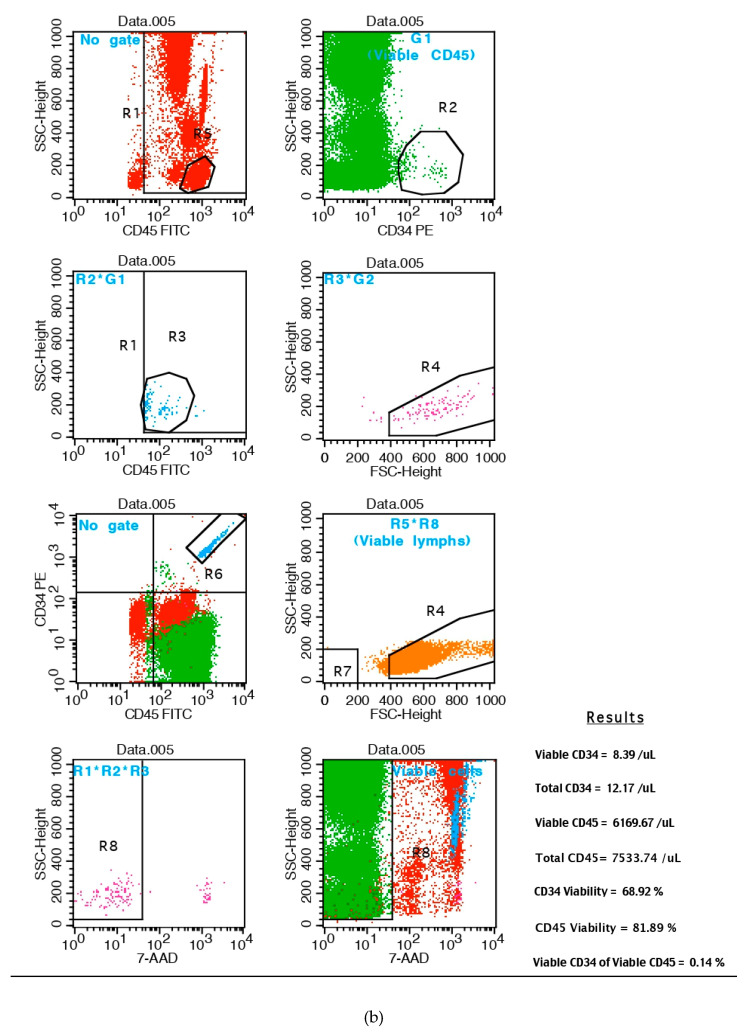

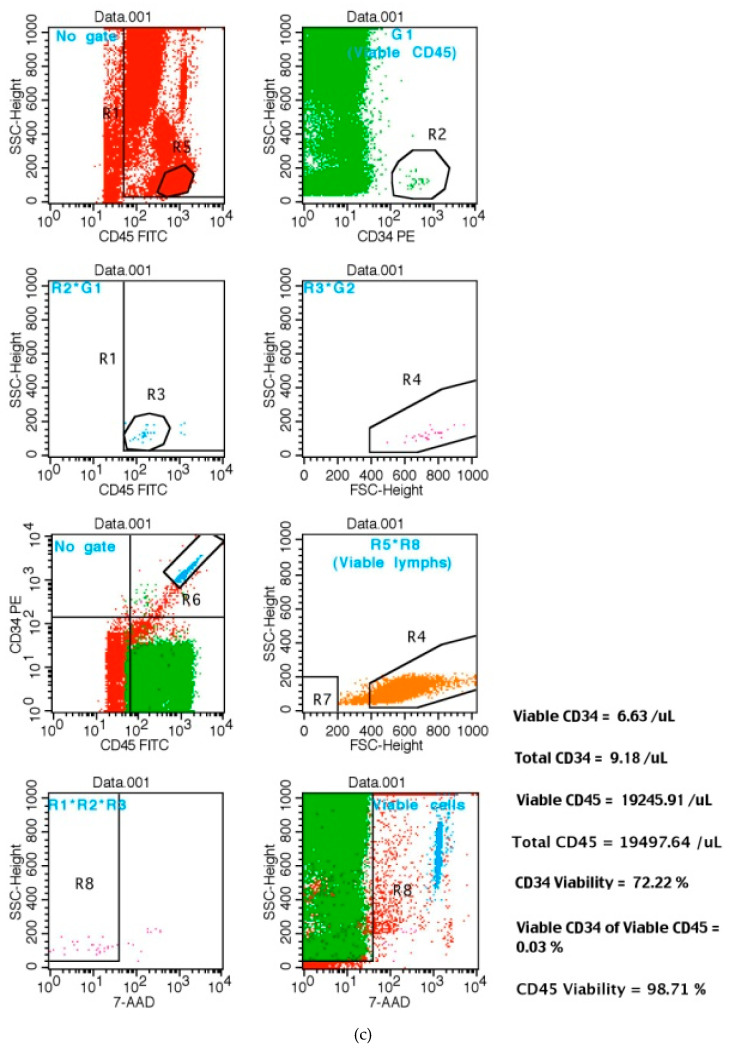

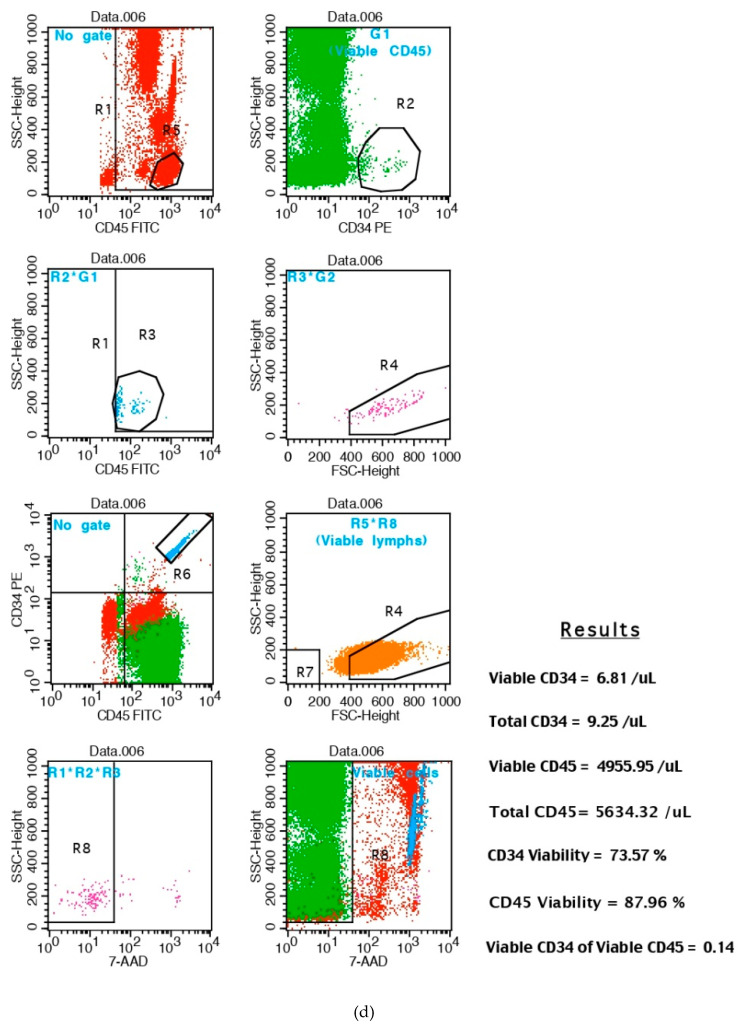
Representative flow cytometry for CD34+ peripheral cells (conts) in study groups. (**a**) Peripheral CD34+ cell (counts) in controls; (**b**) Peripheral CD34+ cells (counts): short-term AFA consumption over 48 consecutive hours; (**c**) Total number of peripheral CD34+ in subjects over 48 h of curcumin administration; (**d**) Effects of long-term nutritional supplementation on total number of CD34+ peripheral cells. Asterisks * are events of data (CD34+ cell counts) within defined regions (R and number) and gates (G and number) for flow cytometry analysis (BD FACSCalibur). The R1*R2*R3 are measured regions for total CD34+ cells (counts) by the BD CellQuest software.

The follows graphs show the representative flow cytometry for CD34+ HSC markers in controls (Figure 6a) as well as in treated-groups (Figure 6b–d).

**Figure 7:**
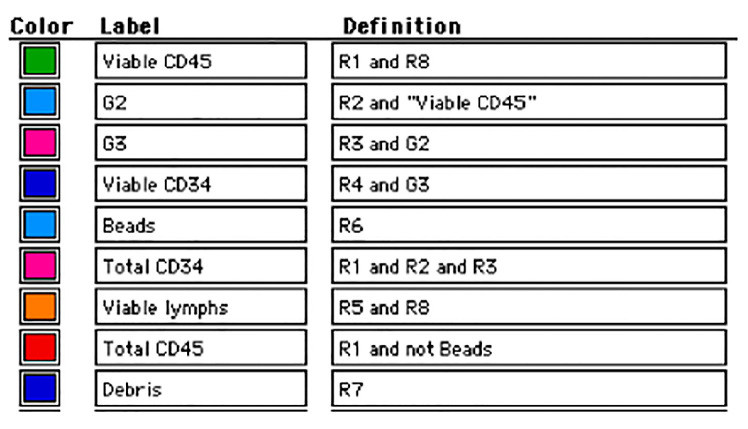
Gate definitions for BD Cell Quest software.

The plots and gating strategy template for BD CellQuest (software for flow cytometry analysis) are automatically showed in the next figure (Figure 7). 

## 4. Discussion

We compared the total number of peripheral CD34+ cells after short-term or long-term nutritional supplementation in healthy subjects with curcuminoids, glycosinolate of sulforaphane, and AFA bluegreen algae extract; the formulation contains several antioxidants, such as curcumin and sulforaphane, which could synergically contribute to increase HSC mobilization. Our findings concur with the protective effects that have been shown in certain antioxidants in hematopoietic-treated cells with resveratrol [17], methoxytryptamine-*α*-lipoic acid [18], chlorophyllin [19], analogues of resveratrol [20], and 5-Methoxytryptamine-*α*-lipoic Acid [21]. In fact, repair mechanisms could be better when CD34+-HSC number of circulating stem cells are accurate [5]. Our findings and these indirect evidences support a role of certain antioxidants as enhancers of HSC mobilization by increasing the total number of CD34+-HSC peripheral cells. Notably, the short-term supplementation with AFA bluegreen algae extract increased the total number of CD34+ peripheral cells after two days of consumption. This enhanced HSC mobilization by AFA, which concurs with a rapid and selective mobilization of specific peripheral stem cell observed after 2 h of polyphenol-rich extract (*Hippophae*) supplementation in healthy patients. However, the total number of CD34+-HSC peripheral stem cells did not differ between treated-patients and non-supplemented subjects. The short AFA bluegreeen algae supplementation increased the total number of circulating CD34+ cells in our study, which concur with enhanced HSC mobilization observed in *cynanophyta Aphanizomenon flos-aquae*-treated subjects; this cyanophyta induces beneficial effects in severe human diseases like cardiomyopathy, stroke, diabetes, rheumatoid arthritis, kidney failure and Parkinson disease [22]. Conversely, the total number of peripheral CD34+ cells are not significant over 48 h of curcumin administration in our study. Interestingly, N-Acetil-Cystein (NAC) antioxidant treatment improved human HSC engraftment and multilineage hematopoietic differentiation in NOD/SCID/IL2r*γ*^−/−^ compromised mice. Compared with control mice, NAC-treated recipient mice had a 10.8-fold increase in hematopoietic engraftment in the injected tibiae [23,24]. The synergic glycosinolate of sulforaphane and curcumin (liquid form) also increased the total number of circulating CD34+- HSC cells after seven consecutive days or 38 days of administration. Thus, synergies between active principles form plants could enhance HSC mobilization in healthy subjects without adverse effects. 

Certain nutraceuticals could act as stem cells mobilizers in rodent models of hematological disease [25,26]. Curcuminoids could contribute to increase HSC mobilization in patients. Interestingly, working memory and attention were improved in curcumin-treated non-demented patients, compared with placebo, 1 h after curcumin administration [12,27,28]. Short-term curcumin-administration did not affect the total number of peripheral CD34+ cells in our study. In general, the curcumin presentations have low bioavailability [29], limiting the clinical efficacy. Pipperine enhances the bioavailability of curcuminoids (powder form) by 2000%; however, HSC mobilization did not differ in healthy subjects after 48 h of curcumin supplementation as compared with their controls. Conversely, the total number of CD34+ peripheral cells increased after seven consecutive days or 38 days of curcumin administration in healthy subjects. We administered curcuminoids over 38 consecutive days, since curcumin was administered over 30 days in non-demented patients or Alzheimer-treated patients [28]. The increased number of peripheral CD34+-HSC cells observed after 38 consecutive days of nutritional supplementation in our study concur with improvements on working memory demonstrated in non-demented subjects after 1 month of curcumin treatment [11]. The synergy between active principles from plants (curcuminoids, glycosinolate of sulforaphane, AFA algae) could contribute to mobilize HSC cells in healthy subjects. In addition, long-term nutritional supplementation with algae extract (*Chlorella or Fucus*) is safe and non-toxic for humans [30]. In fact, glycosinolate of sulforaphane was able to increase neural progenitor cells migration in vitro [31]. Although the precise mechanism by which glycosinolate of sulforaphane could mobilizates HSC cannot be elucidated in our study, it is known that Nrf-2 transcription factor contributes to HSC mobilization [4]; moreover, glycosinolate of sulforaphane [32] or curcumin [33] can activate this transcription factor. Notably, vitamin A and vitamin E have been used to promote beneficial effects in patients for after bone marrow transplantation, whereas vitamin C supplementation can alleviate the functional decrease in lead-exposed HSCs [4]. Curcumin in liquid form is encapsulated with vitamin C in the used formulation. This CD34+ HSC induced-mobilization after 38 consecutive days of supplementation concur with better hematological function reported after antioxidant treatment such as NAC [17,26,34]. 

The synergic administration of active principles from plants could contribute to enhance HSC mobilization in subjects. The antioxidant Phycocianin, a green compound from Aphanizomenos AFA algae, has anti-inflammatory effects [35]. AFA algae contains a polysaccharide fraction (Immolina), which increases the expression of chemotactic cytokines (chemokines) [36]. Chemokines are chemotactic cytokines involved in inflammation, apoptosis, and neuronal progenitor cell migration, among other processes [37]. The interaction between CXCR4 chemokine receptor and, its ligands, Stromal cell-derived factor 1 alpha (SDF 1 alpha) play an important role in hematology [38]. In fact, AMD-3100 (a CXCR4 blocker) treatment mobilizes HSC [39]; the percentage of HSC mobilized cells after curcumin plus AFA/sulforaphane treatment in healthy subjects were equivalent to the number of HSC mobilized cells after 6 h of AMD-3100 treatment in non-human primates [39]. These chemokines promote an HSC-dependent mobilization and certain active principles from plants could regulate these chemokine levels [22,36]. Although chemokines were not evaluated in our study, Larochelle et al. reported that CXCR4 blockade by AMD-3100 increased the total number of CD34+ peripheral cells [39]. The SDF 1 alpha (Stromal Cell Derived factor = CXCL12) chemokine binds to CXCR4 chemokine receptor, which retains stem cells within the bone marrow, because SDF1 alpha is a potent chemoattractant [40]. Although CXCR4/SDF 1 alpha levels were not tested in our study, it is worthy to mention that AFA extract downregulates the expression of CXCR4 on bone marrow stem cells; this downregulation induces a fast stem cell mobilization from the bone marrow into the circulation [39]. AFA bluegreen extract inhibits the fucoidin-induced CXCR4 expression on CD34+ cells from bone marrow but not on the CD34 negative cell line K562 [41]; this feature highlights the role of CD34+ cells in HSC mobilization. The AFA extracts contain a novel ligand for CD62L (L-selectin) [42], which promotes stem cell mobilization in conjunction with down-regulation of the CXCR4 chemokine receptor [39]. These indirect evidences could support that AFA treatment contribute to HSC mobilization in our study. However, AFA extracts alone had only moderate activity in stem cells proliferation assays in vitro [43]. In addition, the white total cells (WBC) or total nucleated cells (TNC) did not differ among treated subjects in the present study.

Curcumin also contributes to the differentiation of some stem cells [44] and promotes apoptosis in leukemia cells in vitro [45]. It appears that certain nutrients, vitamins, and antioxidants such as flavonoids could play roles in maintaining the self-renewal of stem cells and could also stimulate the proliferation and differentiation of progenitors, which are required for the replacement of mature cells in the blood and tissues [46,47,48]. Because certain natural compounds have been found inhibit the growth of tumor cells lines, the synergic supplementation with antioxidants could promote the normal growth of stem cells needed for healing (for example) while also reducing tumorogeneicity potential [44]. These antioxidants could also push normal HSC stem cells into desired phenotypes specific for certain disorders [46,47,48,49]. The synergic effect between antioxidants (such as blueberry, green tea, catechin, carnosine, and vitamin D3) can act to promote healing via an interaction with CD34+ from human hematopoietic stem cells in vitro [50]. 

Our findings are promising since a link between a lower number of circulating stem cells and degenerative diseases were established with Alzheimer disease, cardiovascular diseases, atherosclerosis, diabetes, rheumatoid arthritis, muscular dystrophy, etc. (for review, consult Drapeau et al. [22]). The optimal concentration of the nutraceutical required is a crucial factor for inducing hematopoietic stem cells mobilization in patients. These active principles from plants could have a great potential for broad clinical applications of HSC [35] in diabetes, Alzheimer, and age-related disorders; these pathologies are associated with low HSC cell numbers [48,49,50]. 

## 5. Conclusions and Further Considerations

The short-term consumption of AFA algae extract after 48 h of consumption was associated with a rapid and highly selective enhanced HSC mobilization by increasing the total number of peripheral CD34+-HSC stem cells in healthy subjects as compared with their basal levels (before taking any supplementation) as well as placebo-treated subjects. However, the total number of peripheral CD34+ cells did not differ after 48 consecutive hours of curcumin supplementation (two different formulations). Collectively, the long-term supplementation (curcumin, glycosinolate of sulforaphane, and AFA bluegreen algae) enhanced hematopoietic stem cell mobilization (HSC); the active principles from plants increased the total number of CD34+ peripheral cells in healthy subjects without effect at short times (except with AFA algae administration). The total number of cells (TNC) or white blood cells (WBC) did not differ among subjects in our study. These nutraceuticals may have a great potential for broad clinical applications of HSCs. However, efficacy of a specific antioxidant for BM transplantation will largely depend on the disease type/stages and donor cell types/doses of stem cells. Further studies will evaluate whether CXCR4/SDF-1 alpha chemokines could be differentially regulated in short-term or long-term treated patients with active principles from plants.

## Figures and Tables

**Table 1:** Concentration of antibodies for flow cytometry.

CD45	Concentration 12.5 (μg/mL)
CD34	10
7-AAD	45–65

**Table 2:** Total percentage of CD34+ cells that are also CD45+ in healthy patients.

Study Group	Total Percentage of CD34+ Cells That Are also CD45+ Cells
Placebo (*n* = 7)	0.046 ± 0.049
Control (liquid, *n* = 5)	0.046 ± 0.051
Control (powder, *n* = 5)	0.056 ± 0.0068
Cur 48 h (powder, *n* = 5)	0.066 ± 0.0068 *
Cur 48 h (liquid, *n* = 5)	0.068 ± 0.01
Cur 7 days (*n* = 5)	0.048 ± 0.0031
Cur 38 days (*n* = 5)	0.066 ± 0.0068
Cont (Algae, *n* = 5)	0.053 ± 0.043
AFA (48 h, *n* = 5)	0.1 ± 0.0005 * # * *p* < 0.05 vs. control (algae)
	* *p* < 0.05 vs. control (powder) # *p* < 0.05 vs. placebo

Results are the mean values for CD34+ and CD45+ positive cells (at the same time) ± SEM. Differences are considered statistically significant when *p* < 0.05. * *p* < 0.05 vs. controls (before taking any nutritional supplementation). # *p* < 0.05 vs. placebo. Cont: controls. * *p* < 0.05 vs. control (algae) or control (powder curcumin).

**Table 3:** Absence of differences for TNC or WBC levels in healthy patients.

Study Group	TNC (×10^8^)	WBC (×10^6^/mL)
Placebo (*n* = 7)	5.32 ± 0.98	3.08 ± 0.37
Control (powder *n* = 5)	5.8 ± 0.35	3.3 ± 0.3
Control (liquid, *n* = 5)	6.2 ± 0.75	3.08 ± 0.17
Cur 48 h (powder, *n* = 5)	6.21 ± 0.3	2.9 ± 0.43
Cur 48 h (liquid, *n* = 5)	6.32 ± 0.28	2.54 ± 0.43
Cur 7 days (*n* = 5)	5.52 ± 0.29	3.07 ± 0.17
Cur 38 days (*n* = 5)	5.89 ± 0.19	3.16 ± 0.35
Cont (Algae, *n* = 5)	5.7 ± 0.42	3.46 ± 0.13
AFA (48 h, *n* = 5)	6.21 ± 0.3	2.96 ± 0.3
